# Biosynthesis of Osmoregulated Periplasmic Glucans in *Escherichia coli*: The Phosphoethanolamine Transferase Is Encoded by *opgE*

**DOI:** 10.1155/2013/371429

**Published:** 2013-10-22

**Authors:** Sébastien Bontemps-Gallo, Virginie Cogez, Catherine Robbe-Masselot, Kevin Quintard, Jacqueline Dondeyne, Edwige Madec, Jean-Marie Lacroix

**Affiliations:** ^1^Unité de Glycobiologie Structurale et Fonctionnelle, UMR CNRS 8576, IFR 147, Université des Sciences et Technologies de Lille, 59655 Villeneuve d'Ascq Cedex, France; ^2^Université Lille Nord de France, Lille, France

## Abstract

Osmoregulated periplasmic glucans (OPGs) are oligosaccharides found in the periplasm of many Gram-negative bacteria. Glucose is the sole constitutive sugar and this backbone may be substituted by various kinds of molecules depending on the species. In *E. coli*, OPG are substituted by phosphoglycerol and phosphoethanolamine derived from membrane phospholipids and by succinyl residues. In this study, we describe the isolation of the *opgE* gene encoding the phosphoethanolamine transferase by a screen previously used for the isolation of the *opgB* gene encoding the phosphoglycerol transferase. Both genes show structural and functional similarities without sequence similarity.

## 1. Introduction

**Figure 1:**
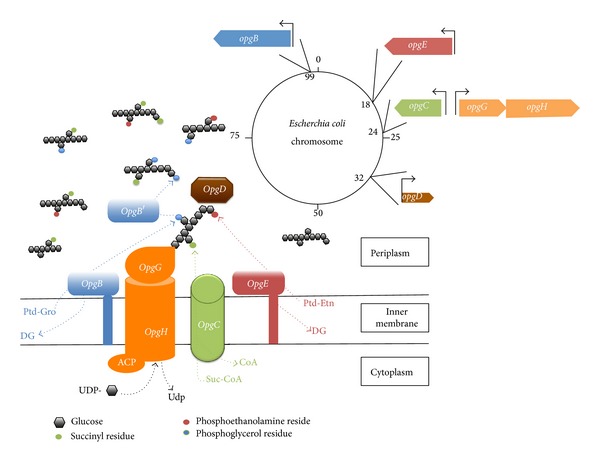
OPGs biosynthesis in *E. coli*: working model. For details, see Section 1. On top right, chromosomal location of *opg* genes. Ptd-Gro: phosphatidylglycerol, Ptd-Etn: phosphatidylethanolamine, DG: diglyceride, Suc-CoA: succinyl-coenzyme A, and CoA: coenzyme A.

Osmoregulated periplasmic glucans (OPGs), formerly membrane derived oligosaccharides are oligosaccharides accumulated in the envelope of many Gram-negative bacteria in media of low osmolarity. They belong to the common virulence factors found in phyto- and zoo pathogen of many Gram-negative bacterial species. In addition, OPGs control motility and secretion of exopolysaccharides in several species [7–9]. Glucose is the sole constitutive sugar and this glucosidic backbone may be substituted by various substituents depending on the species [10]. In *Escherichia coli*, OPG backbone is synthesized by the *opgGH* products (Figure 1) [11]. Total loss of OPGs reduced motility and increased exopolysaccharides synthesis and they are phenotypes associated with inactivation of *opgG* or *opgH* genes. OpgH is a transmembrane glucosyl-transferase catalyzing, with acyl carrier protein (ACP) as a cofactor and UDP-glucose as a substrate, the synthesis of a backbone constituted of linear *β*-1,2-linked glucose units. OpgG is a periplasmic glucosyl transferase branching glucose units on this linear backbone by *β*-1,6-linkages [10]. This backbone is highly substituted by phosphoglycerol residues, and to a less extent by phosphoethanolamine residues, derived from membrane phosphatidylglycerol and phosphatidylethanolamine, respectively, and by succinyl residues (Figure 1) [10]. Two phosphoglycerol transferases catalyze the substitution by phosphoglycerol and are encoded by the same *opgB* gene [12]. The phosphoglycerol transferase I is anchored to the inner membrane but a large periplasmic catalytic domain transfers phosphoglycerol residues from phosphatidylglycerols to OPG molecules. The phosphoglycerol transferase II is a periplasmic soluble enzyme resulting from the liberation of the periplasmic catalytic domain of the former one and catalyzes the transfer of phosphoglycerol residues from one OPG molecule to another. Both phosphoglycerol transferases can transfer phosphoglycerol to artificial *β*-glucoside acceptors such as arbutin leading to enhanced turnover of phosphatidylglycerol. This was the basis of the selection of the *mdoB* mutants. In a *dgk* strain grown in media of low osmolarity containing arbutin, accumulation of diacylglycerol occurred in membrane to a toxic level and cell growth slowed abruptly. In a double mutant *dgk opgB*, diacylglycerol accumulation decreased and growth resumed [13]. The succinyl-transferase is encoded by the *opgC* gene and is an intrinsic transmembrane protein catalyzing the transfer of succinyl residues probably from succinyl-CoA to OPG molecules. In an *opgB* strain, anionic character of OPGs is only provided by succinyl residues since phosphoethanolamine is a neutral substituent. This was the basis of the isolation of the *opgC* mutant which was isolated by screening an *opgB* strain using the severe difference of migration within a thin layer chromatography between neutral and anionic OPGs [11].

In this paper, we describe the isolation of a new gene, *opgE*, needed for substitution of OPGs by phosphoethanolamine in *E. coli* by using a similar selection protocol previously described for isolation of the *opgB* mutant.

## 2. Material and Methods

### 2.1. Bacterial Strains, Media, and Growth Conditions

*E. coli* strains and bacteriophage are listed in Table 1. Bacteria were grown with vigorous shaking at 37°C in Luria-Bertani broth (LB) [14] unless, otherwise, it is indicated. Solid media were obtained by adding agar at 15 g/liter. For motility measurement, highly wet substrate is required and agar was added to LB medium at 3 g/liter (swimming medium). 10^7^ bacteria were spotted onto this swimming medium in a volume of 5 *μ*L and the intensity of the halo of swimming was measured after 24 h of incubation at 37°C. When low osmolarity medium was required, LB without NaCl (LB-N) was added and used [11]. For exopolysaccharides secretion, bacteria were streaked onto the SOB-Glycerol solid medium [15]. Exopolysaccharides secretion level was measured by intensity of the slimy aspect of bacterial colonies (also called mucoidy) after 30 h of growth at 30°C. Antibiotics were used at the following concentrations: ampicillin and kanamycin at 50 *μ*g/mL and chloramphenicol at 25 *μ*g/mL. X-Gal (5-bromo-4-chloro-3-indolyl-*β*-D-galactopyranoside) were used at a concentration of 40 *μ*g/mL. Arbutin was used at a concentration of 90 mM.

### 2.2. Recombinant DNA Techniques

Standard procedures were performed for genomic and plasmid DNA extractions. gDNA purification was performed with the Nucleospin tissue (Macherey-Nagel). DNA purification was performed with the Nucleospin miniprep (Macherey-Nagel). Restriction enzymes (fermentas), T4 DNA ligase (fermentas), and Taq polymerase (fermentas) were used according to the manufacturer's recommendations.

### 2.3. Transduction and Transformation

Transduction with phage P1*vir* and transformation by the rubidium chloride technique of *E. coli* were carried out according to Miller [14].

### 2.4. Tn*5* Transposon Mutagenesis

Random Tn*5* mutagenesis was performed with *λ*NK467 (Table 1) according to standard procedures [16]. Bacterial strain NFB775 was grown in LB liquid medium until mid log phase at 37°C in LB medium. Phage *λ*NK467 was added to bacteria at a multiplicity of infection (MOI) of 1 and incubated without shaking for 30 min. to allow adsorbtion of phages and entry of phage DNA in bacterial cells. Bacteria (10^9^) were then plated onto LB plates containing kanamycin and incubated for 48 h at 42°C. The kanamycin resistant clones obtained were only the result of transposition of Tn*5* from *λ*NK467 to the bacterial DNA since in these conditions, *λ*NK467 became a suicide vector because it cannot induce neither lytic cycle (mutation in the replication genes *Oam* and* Pam*) nor lysogeny (thermosensitive mutation of the repressor CI^857^).

### 2.5. Tn*5* Insertion Localization

Tn*5* transposition insertions were localized on the *E. coli* chromosome by inverse PCR using Tn*5*CEcoRI and Tn*5*DHindIII primers (Table 1) after digestion of the chromosomal DNA with taqI.

### 2.6. Cloning of the *opgE* Gene and Construction of the *opgE::cml* Mutation

Plasmids and primers designed for PCR are listed in Table 1. A 2073 bp DNA fragment containing the *opgE* gene was amplified by PCR (foropgE and revopgE primers) from genomic DNA. For cloning of the *opgE* gene, this PCR product was digested by EcoRI and HindIII, cloned into pUC18Not and digested by the same enzymes and transformed into competent *E. coli* cells to give pNF752. For construction of the *opgE::cml* mutation, the chloramphenicol cassette used for gene inactivation was released from pNFCml [5] after digestion by EcoRV and was inserted into the unique HpaI restriction site of the 2073 bp PCR DNA fragment containing *opgE*. The resulting DNA fragment was introduced into competent *E. coli* cells containing the pKD46 plasmid allowing homologous recombination by expression of the *red* recombinase gene products [6]. The accurate insertion of the mutation was controlled by PCR using genomic DNA of the resulting *opgE::cml* mutant as a template and upopgE and C1 and downopgE and C2 as primers.

### 2.7. Extraction of OPGs

Bacteria were grown overnight in LB without NaCl (200 mL). Bacteria were collected by centrifugation at 4°C for 15 min at 8,000 ×g. Cell pellets were resuspended in 20 mL of distilled water and extracted with 5% trichloroacetic acid (TCA). The TCA extracts were neutralized with ammonium hydroxide 10% and concentrated by rotary evaporation. The resulting material (2 mL) was then fractionated by gel filtration on a Bio-Gel P-4 column (Bio-Rad, 55 by 1.6 cm) equilibrated with 0.5% acetic acid. The column was eluted in the same buffer at a flow rate of 15 mL h^−1^, and fractions of 1.5 mL were collected. Presence of sugar in each fraction was determined colorimetrically by the anthrone reagent procedure. Fractions containing OPGs were pooled and total content was determined by the same procedure [17]. For mass spectrometry analysis, OPGs were subsequently desalted in water on a Bio-Gel P-2 column (Bio-Rad, 90 by 1.6 cm) and fractions containing OPGs were lyophilized.

### 2.8. Mass Spectrometry

All mass spectra were acquired on a Voyager Elite (DE-STR) reflectron time-of-flight (TOF) mass spectrometer (Perseptive Biosystems, Framingham, MA), equipped with a pulsed nitrogen laser (337 nm) and a gridless delayed extraction ion source. Oligosaccharide samples were analyzed in delayed extraction mode using an accelerating voltage of 20 kV, a pulse delay time of 200 ns, and a grid voltage of 66%. Detector bias gating was used to reduce the ion current for masses below 500 Da. Between 100 and 200, scans were averaged for each mass spectrum. Oligosaccharide alditols were cocrystallized with 2,5-dihydroxybenzoic acid (DHB) as matrix (10 mg mL^−1^ of DHB in methanol/water (50/50) containing 0.1% trifluoro acetic acid (TFA)). For all measurements, the “dried droplet” preparation technique was used. Typically, 1 mL of the matrix was mixed on-target with 1 *μ*L of water-dissolved oligosaccharides and allowed to dry under an air stream. They were analyzed in positive ion mode.

## 3. Results and Discussion

### 3.1. Arbutin Is a Substrate for the Phosphoethanolamine Transferase

**Figure 2:**
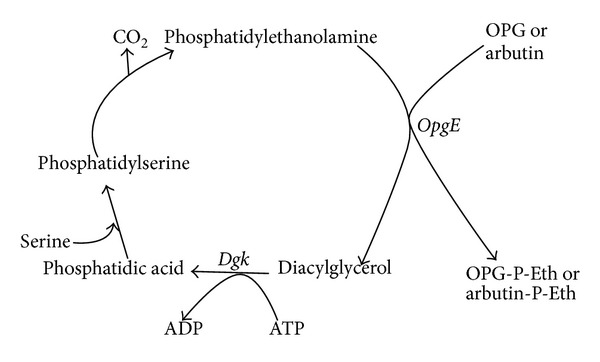
Phosphoethanolamine cycle. This cycle shows that in a *dgk* strain, larger amounts of diacylglycerol will accumulate when growth occurrs in medium supplemented with arbutin. P-Eth: phosphoethanolamine and OPG: osmoregulated periplasmic glucans.

Arbutin is an artificial substrate for the phosphoglycerol transferase and may be also substrate of the phosphoethanolamine transferase since they catalyze similar reactions (transfer of polar head from phospholipids to OPGs). In a *dgk* strain, arbutin resistance was used to isolate the *opgB* gene (see Section 1) and similar procedure could be used to isolate mutant devoid of the phosphoethanolamine transferase. The screen could be used only if severe differential growth of bacterial colonies was shown between the NFB775 grown without arbutin (normal growth) or with arbutin (slow growth resulting from accumulation of toxic diacylglycerol) (Figure 2). The NFB775 (*opgB dgk*) strain, synthesizing OPGs substituted with succinyl and phosphoethanolamine residues but devoid of phosphoglycerol residues, was grown at 37°C in LB-N liquid medium overnight. Appropriate dilution of the culture was performed and the same volume of dilution was spotted onto 6 LB-N medium plates and onto 6 LB-N medium plates with Arbutin 90 mM being added. These 12 plates were incubated at 37°C and colony forming units (CFU) were numbered in the same 12 plates after 24 h, 48 h, and 72 h of incubation. In LB-N medium, an average of 243 CFU of normal size were numbered after 24 h of incubation and 260 CFU were counted after 48 h or 72 h of incubation. In LB-N medium with arbutin, an average of 15 very small CFU were hardly detected after 24 h of incubation, 146 small CFU after 48 h of incubation, and 207 medium CFU after 72 h of incubation. Thus, growth is severely impaired in NFB775 grown in LB-N with arbutin added as compared to the same medium without arbutin added and colony size were still smaller after 72 h of growth in medium containing arbutin as compared to colonies grown 24 h in medium without arbutin added. Thus, isolation of arbutin resistant mutants could be performed. Because the *dgk* strain tested was devoid of phosphoglycerol transferase (*opgB*), one can imagine that arbutin is also a substrate for phosphoethanolamine transferase stimulating its activity, increasing phosphatidylethanolamine turnover leading to accumulation of diacylglycerol toxic for bacteria (Figure 2) and that at least one kind of arbutin resistant mutants could result from the inactivation of the gene encoding the phosphoethanolamine transferase.

### 3.2. Screening of an *opgE*::Tn*5* Mutant

Random transposon mutagenesis with Tn*5* was performed on the NFB775 strain (see Section 2). 30000 clones kanamycin resistant were collected and divided into 20 pools of 1250 clones. One LB-N plate containing 90 mM arbutin was used to plate independently each of the 20 pools and incubated at 37°C. The first 30 clones that appeared on these plates (average of 1.5 clone per plate) were collected after 24 h, purified, and grown in LB-N liquid medium. One LB-N plate containing 90 mM arbutin was used to plate independently 200 CFU of each of the 30 cultures and incubated at 37°C. The 5 plates harboring bacterial colonies growing faster than the others were selected and one clone isolated from each of the 5 plates was further studied. The 5 genes disrupted by transposon were located on the *E. coli* chromosome (see Section 2). The first two transposons were inserted in the intergenic DNA region lying between the *malK* and *lamB* genes of the *malK lamB malM* operon of the maltose regulon, thus inactivating *lamB* and *MalM*. The third transposon was inserted within the *yidL* gene encoding a putative AraC regulatory protein. The fourth transposon was inserted within *fucI* encoding a L-fucose isomerase. The fifth transposon was inserted within *ybiP* encoding a putative inner membrane protein with a sulfuric ester hydrolase and transferase activities domains (accession number Ecocyc G6418). These two domains were also found on the OpgB protein (accession number Ecocyc EG12591). Thus, we decided to further characterize the *ybiP* mutant clone.

### 3.3. The *opgE *Gene Encodes the Phosphoethanolamine Transferase

**Figure 3:**
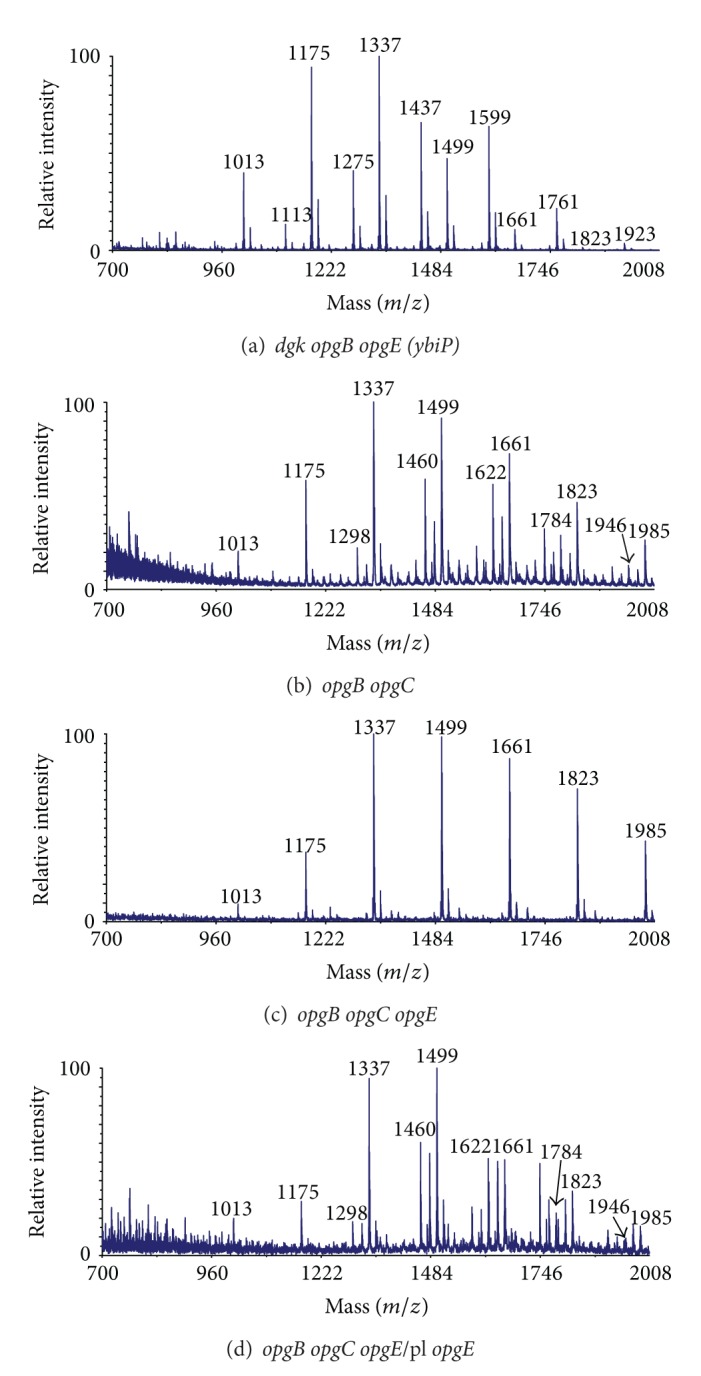
Mass spectra of purified OPGs. MALDI mass spectra acquired in positive ion mode on purified OPGs extracted from (a) *dgk opgB opgE *(*ybiP*) strain, (b) the *opgB opgC *strain, (c) the *opgB opgC opgE *strain, and (d) the *opgB opgC opgE *strain complemented with the plasmid harboring the *opgE* gene (pl *opgE*). The [M+Na]^+^ molecular ions are indicated on the top of each peak. As examples, arrows indicated the [M+Na]^+^ molecular ions of DP11 and DP12 substituted by one phosphoethanolamine residue.

Mass Spectrum of OPGs extracted from the *ybiP* mutant clone grown in LB-N medium was performed. An increment of 100 Da, corresponding to substitution of OPGs by succinyl residues was observed for several molecular ions. No increment of 123 Da, corresponding to phosphoethanolamine substitution of OPGs, was observed (Figure 3). This strongly suggests that *ybiP*, renamed *opgE*, encodes the phosphoethanolamine transferase. To confirm that *opgE* encodes the phosphoethanolamine transferase, *opgE* was cloned into pUC18Not (pNF752) and inactivated on the *E. coli* chromosome by reverse genetic (*opgE*::*cml*) (see Section 2). Mass spectra of OPGs extracted from NFB2228 (*opgB opgC*), NFB4548 (*opgB opgC opgE*), and NFB2231 (NFB4548/pNF752) were compared (Figure 3). The sizes of OPGs varied from 6 to 12 residues of glucose, as illustrated on MS spectra by the [M+Na]^+^ molecular ions at *m/z* 1013, 1175, 1337, 1499, 1661, 1823, and 1985, respectively. As expected, OPGs were neither substituted by succinyl residues nor by phosphoglycerol residues in these mutants. OPGs extracted from NFB4548 strain, in which *opgE* has been inactivated, were not substituted by phosphoethanolamine residues (Figure 3). On the contrary, in NFB2228 and NFB2231 strains, in which *opgE* is functional, a mass increment of 123 Da was observed for OPGs, with, for example, molecular ions at *m/z* 1298, 1460, 1622, 1784, and 1946 (Figure 3). This mass increment corresponded to the substitution of OPGs by phosphoethanolamine residues and confirmed that *opgE* encodes the phosphoethanolamine transferase. Taken together, these results suggest that arbutin is not only a substrate for the phosphoglycerol transferase but also for the phosphoethanolamine transferase (Figure 2). Nevertheless, the arbutin resistance is probably a more complex phenomenon than previously thought. The reason for the arbutin resistance of the 4 other mutants is difficult to explain and is probably not related to OPGs suggesting that arbutin affects several different cellular processes.

### 3.4. Sequences Analysis of *opgE*

**Figure 4:**
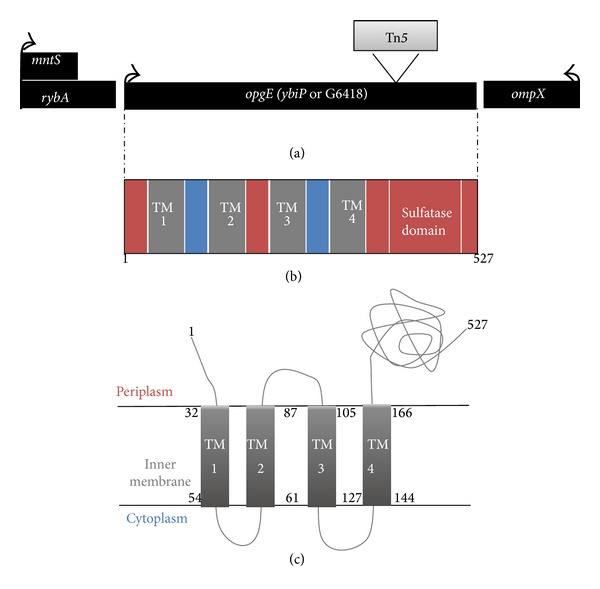
(a) Genetic organization of the *opgE* region. Chromosomal position of the *opgE* gene and Tn*5* insertion of the *opgE* mutant isolated after the arbutin screen are indicated. (b) Predictive organization of the OpgE protein. Transmembrane segments are in grey, cytoplasmic regions are in blue, and periplasmic regions are in red and sulfatase domain within the last periplasmic region is shown. (c) Predictive model of the OpgE protein. First and last amino acids of the OpgE protein are indicated and first and last amino acids of each transmembrane (TM) segment are indicated.

*opgE* is an operon constituted of one gene transcribed counterclockwise. The 1584 bp open reading frame begins at 851 820 bp and ends at 850 237 bp on the *E. coli* K12 chromosome and encodes a 527 amino acid protein (Figure 4) of 59 707 Da. The TMHMM2.0 algorithm [18] allowed the prediction of four transmembrane spanning segments in the 166 first amino acids and a periplasmic domain in the 361 following amino acids. The 217 to 483 amino acid sequence form a 226 amino acids catalytic domain with sulfuric ester hydrolase and transferase activities. Arbutin cannot cross the inner membrane of *E. coli* K12 strongly suggesting a periplasmic location of this catalytic domain. The OpgB protein shows a very similar organization [12] with 3 or 4 transmembrane segments in the 162 first amino acids followed by a periplasmic domain containing a 285 amino acids sulfuric ester hydrolase and transferase activities (amino acids 163 to 448) [12]. Despite the similarity between structure and function (except the donor phospholipid) of these two enzymes, no significant similarity was observed between the OpgB and OpgE amino acids sequences even in the hydrolase and transferase catalytic domains.

### 3.5. Phenotypes of *E. coli* Strains Devoid of Substituents

**Figure 5:**
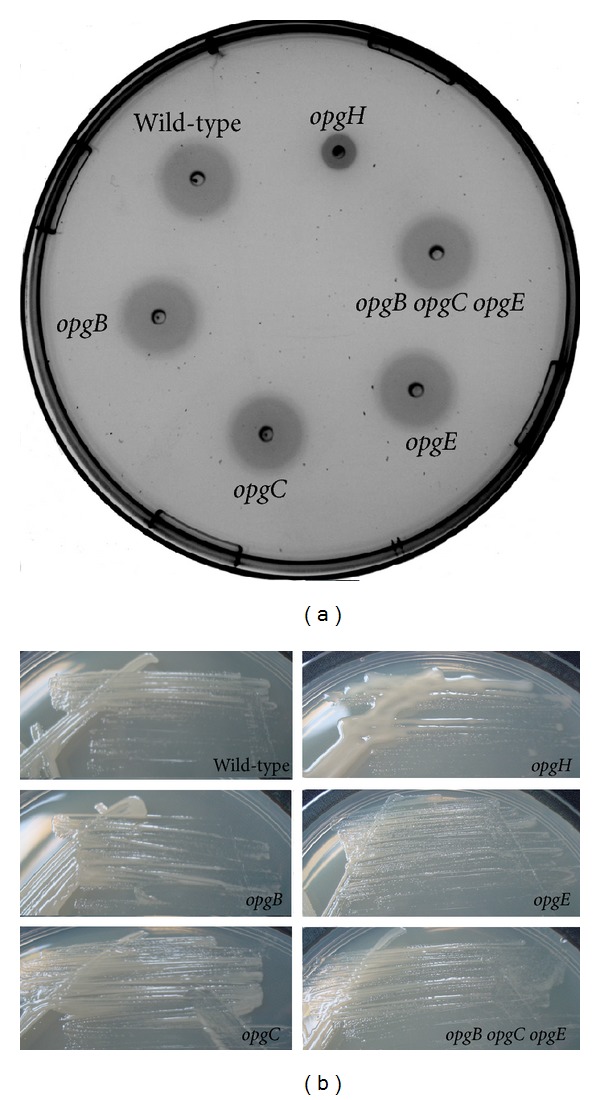
Motility and mucoidy analysis of *opg* mutants of *E. coli*. (a) Motility: strains (10^7^ CFU) were spotted onto swarming medium containing 0,3% agar. The plates were incubated at 37°C and motility was examined after 24 h of incubation. (b) Mucoidy: The same strains were streaked on SOBG medium. The plates were incubated at 30°C and mucoidy was examined after 30 h of incubation.

The main phenotypes of mutant strains devoid of OPGs backbone (i.e., *opgG* or *opgH*) are loss of motility and increased exopolysaccharides synthesis (see Section 1). We decided to test these phenotypes in strains devoid of one or all kinds of substitution on OPGs backbone. For motility measurement, *opgB* (NFB732), *opgC* (NFB1887), *opgE* (NFB4576), and *opgB opgC opgE* (NFB4548) strains were spotted on swimming agar (see Section 2) for 24 h together with the wild type (JM83) and the *opgH* (NFB216) strains as controls. As expected for the two controls, the wild-type strain was motile while motility was severely reduced for the *opgH* strain. As observed for the wild-type strain, all the mutant strains tested were motile (Figure 5). For exopolysaccharides synthesis measurement, the same strains were plated onto SOB-Glycerol medium. As observed for the wild-type strain, all the mutant strains were non mucoid indicating no increase in exopolysaccharides synthesis except for the *opgH* mutant strain where mucoidy was increased indicating an increase in exopolysaccharides synthesis (Figure 5). Thus, no classical phenotype found in mutants devoid of OPGs backbone could be associated with mutants only devoid of substituent. Thus, the role of substituent remains to be elucidated.

## 4. Conclusion

The isolation of the *opgE* gene strongly suggests that the minimum set of genes required for a complete biosynthesis pathway of OPGs were characterized (Figure 1). The suggested interactions [10] between several of these enzymes suggest that these enzymes work within a complex which remains to be established. The role and the reason of the difference of substitution depending on the species remain to be established but the absence of phenotype renders this analysis difficult.

## Figures and Tables

**Table 1:** Strains, plasmids, and primers.

Strain, phage, plasmid, or primers	Relevant genotype and/or phenotype	Source or reference
*Escherichia coli *		
JM83	*ara *Δ*(lac-pro) thi rpsL *Φ*80*Δ*(lacZ15) *	[1]
NFB216	JM83* opgH200::*Tn*10 pyrC46 *	[2]
NFB732	JM83* opgB214::*Tn*10 *	[3]
NFB775	*HfrH thi-1 serB28 opgB214::*Tn*10 dgk-6 relA1 *	This study
NFB1887	JM83* opgC1::*Tn*5 *	This study
NFB2228	JM83* opgB214::*Tn*10 opgC::*Tn*5 *	This study
NFB4548	JM83* opgB214::*Tn*10 opgC::*Tn*5 opgE2::cml *	This study
NFB4576	JM83* opgE2::cml *	This study
NFB2231	NFB4548/pNF752	This study

Phage		
*λ*NK467	*b221 * *c1* ^857^ *Oam29 Pam8O rex::Tn5 *(KanR)	N. Kleckner

Plasmids		
pUC18Not	AmpR	[4]
pNFCml	AmpR, CmlR	[5]
pKD46	AmpR lambda Red recombinase expression	[6]

Primer		
Tn5CEcoRI	GTGAATTCACTCCGTTCTCTTGCTCG	This study
Tn5DHindIII	GGGAAAGCTTCCGTTCAGGACGCTAC	This study
foropgE	CCTGAATTCCCGGTGTTGGTTACCGCTTC	This study
revopgE	GGAAAGCTTATGAATGAGTTCAAGAGGTG	This study
upopgE	GGATCGACGGGATTAGCAAG	This study
c1	TTATACGCAAGGCGACAAGG	[6]
downopgE	ACGACTGGGCAAGCATCTAC	This study
c2	GATCTTCCGTCACAGGTAGG	[6]
